# Transplantation of mesenchymal stem cells in a laryngeal carcinoma patient with radiation myelitis

**DOI:** 10.1186/s13287-015-0203-1

**Published:** 2015-11-04

**Authors:** Jun Liang, Fan Wang, Dandan Wang, Huayong Zhang, Cheng Zhao, Shiying Wang, Lingyun Sun

**Affiliations:** Department of Rheumatology and Immunology, the Affiliated Drum Tower Hospital of Nanjing University Medical School, 321 Zhongshan Road, Nanjing, CN 210008 P. R. China

## Abstract

Radiation myelitis is a rather rare but devastating complication following therapeutic irradiation to neoplasms when the spinal cord is included within the radiation field. Symptoms of radiation myelitis with the therapeutic doses of radiation commonly employed are usually delayed and most often appear about 6 to 24 months following irradiation. So far, no treatment has proved satisfactory.

Transplantation of allogeneic mesenchymal stem cells has been a promising therapy strategy for many disorders in the central nervous system, such as multiple sclerosis, neuromyelitis optica, and autoimmune encephalomyelitis. The cell-base therapy has shown to act to limit inflammation of central nervous system, stimulate neurogenesis, protect axons and promote remyelination. But it has not been established as a therapeutic option for radiation myelitis.

In this report, we describe the outcome of allogeneic umbilical cord-derived mesenchymal stem cell transplantation in a patient with laryngeal carcinoma who developed radiation-induced myelitis of his spinal cord with characteristic magnetic resonance imaging changes.

## Introduction

Radiation myelitis, while rare, is one of the gravest complications in radiation therapy. Some reports have attempted to decrease the incidence of radiation myelitis by minimizing the total radiation dose and the fraction size [1, 2]. However, few articles have focused on the therapy of radiation myelitis. Up to now, therefore, the treatment of radiation myelitis consists mainly of its prevention; and when this fails, symptomatic measures are usually employed. Some cases demonstrated dramatic response to high-dose steroid treatment [3, 4], but steroids were shown to obtain temporary remissions or only delay the progression of radiation myelitis for a short period of time [5]. Furthermore, other cases showed that steroids had no influence on the progression of disease [6].

Mesenchymal stem cells (MSCs), a subset of nonhematopoietic cells, possess pluripotent features and can be obtained from different sources such as bone marrow, umbilical cord (UC), UC blood, and adipose tissue of the human body. These cells are able to differentiate into tissues of mesenchymal lineages, including bone, cartilage, fat, tendon, muscle, and stroma [7–11]. Recently, research has shown that MSCs can differentiate into neural stem cells, mature functional neurons, and glial cells [12–15], while no data suggest that MSCs can differentiate into axons. Liu et al. [16], however, have reported that acellular spinal cord scaffold seeded with MSCs is able to bridge a spinal cord cavity and promote long-distance axon regeneration and functional recovery in spinal cord injury rats. The second property of MSCs is their capacity for immunomodulation. Emerging evidence has demonstrated that human MSCs can inhibit, in a dose-dependent manner, the proliferation and cytokine production of many allogeneic immune cells, such as T cells, B cells, natural killer (NK) cells, and dendritic cells [17–19]. MSCs have also been found to release a number of soluble immunosuppressive factors involved in MSC-mediated immunoregulation, such as indoleamine 2,3-dioxygenase, interleukin (IL)-6 and HLA-G5 [20]. In addition, their low immunogenicity—due to their lack of expression of class II major histocompatibility complex and costimulatory molecules—makes MSCs able to escape alloantigen recognition and then unable to activate alloreactive T cells. The third important property of MSCs is their capacity to migrate and home to their target tissues after infusion, like other stem cells. There is a hypothesis that they can home into, and adjust their differentiation pathway to, diverse tissue microenvironments [21]. Animal studies have evaluated that MSCs can migrate into injured spinal cord tissue through cerebrospinal fluid and into the brain though peripheral blood [15, 22]. These properties make MSCs therapeutic potential cells in many chronic inflammatory demyelinating diseases and other central nervous system (CNS) diseases. MSCs have so far been applied successfully in patients with multiple sclerosis, neuromyelitis optica, and ischemic stroke, and in animal models of autoimmune encephalomyelitis [23–27]. In our center, UC MSCs have shown an extraordinarily therapeutic effect in patients with refractory lupus, inflammatory bowel disease, and rheumatoid arthritis [28–31]. All of these data suggest that MSCs may be exerted in the treatment of radiation myelitis.

## Method

A 37-year-old male was diagnosed with laryngeal carcinoma, underwent total laryngectomy, and subsequently received more than 30 fractions of focal area radiotherapy in April 2010, with total radiation doses of 6000 cGy. One year later, the patient complained of right arm numbness and weakness, which progressively extended to both upper extremities and the area below his chest. Magnetic resonance imaging (MRI) of the spine demonstrated an enhanced lesion in the radiation field. Cytology of cerebrospinal fluid was normal. Four months after onset of clinical manifestations, leg weakness worsened resulting in multiple falls and difficulty in walking. Radiation myelitis was considered. Administration of corticosteroids (dexamethasone 10 mg/day for 10 days) in local hospitals partially ameliorated the symptoms. However, 1 month later the patient suffered progressive worsening of lower limb numbness and weakness, and additional neurological symptoms such as absence of bladder sensation and incontinence emerged during tapering to low-dose steroids (prednisone 10 mg/day). During the second period in the local hospital, treatment with the same dose of dexamethasone for 2 weeks, ganglioside, and mecobalamine did not show any beneficial results. The patient was then transferred to our department for further treatment.

**Fig. 1:**
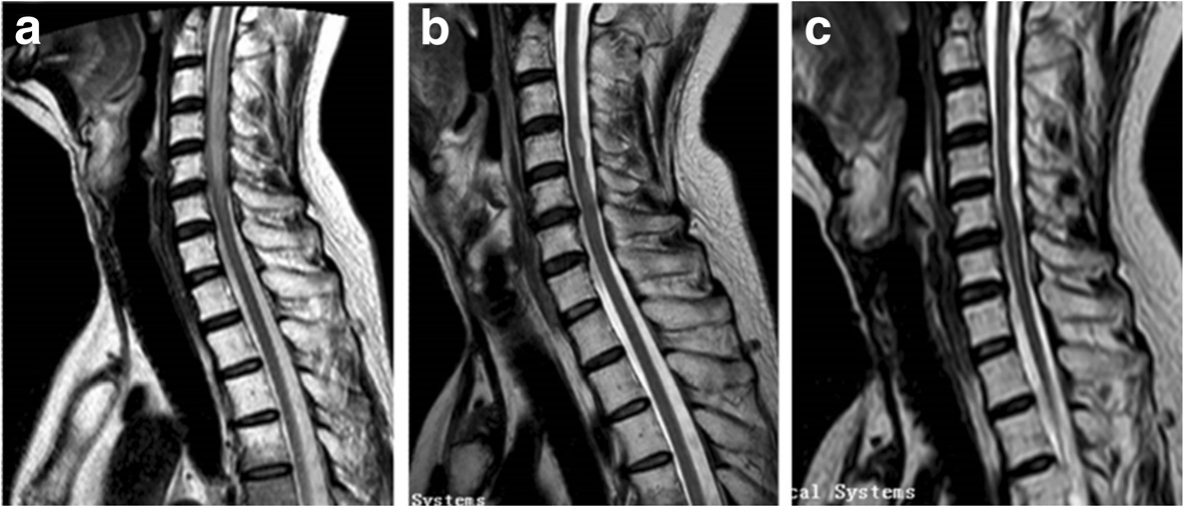
Comparison of MRI T2-weighted images of the spinal cord. **a** MRI before UC-MSCT revealing fresh linear hyperintensities within the swollen cervical cord from C4 to C6. **b** At 6-month follow-up, MRI demonstrated obvious regression of the previous lesions. Swelling of the spinal cord was reduced significantly. **c** At 18-month follow-up, MRI did not show new enhanced lesions in the spinal cord

Examination showed reduced power in four extremities (1 out of 5 on the Medical Research Council scale). Pain and temperature sensations were lost below the T2 level, while vibration and position sense were preserved. The remainder of the patient’s general and neurologic examination was normal. The administration of corticosteroid (methylprednisolone 500 mg/day intravenous pulse therapy) and mecobalamine was started, and omeprazole was also added. However, there were no improvements in muscle strength and bladder function after 5 days of high-dose methylprednisolone, so the dose was tapered based on the underlying adverse effects and ineffectiveness of steroids. A regimen of intravenous methylprednisolone 40 mg/day together with omeprazole, aspirin, and mecobalamine was given on the following days. At the end of second week of admission, an MRI scan of the head and neck was taken. The craniocerebral scan was normal, while radiation myelopathy and radiation osteomyelitis was obvious (Fig. 1a): hyperintense signals were visible within the swollen cord from C4 to C6, with abnormal signal intensity in the vertebral body starting from C2 to T4. We thus turned to umbilical cord-derived mesenchymal stem cell transplantation (UC-MSCT), due to the unresponsiveness of steroid treatment and promising results for MSC transplantation in the treatment of CNS disorders.

UC MSCs were prepared by the Stem Cell Center of Jiangsu Province (Jiangsu Beike Bio-Technology, Taizhou, Jiangsu, China) as described previously with some modification [32, 33]. Fresh UC was obtained from an informed healthy mother in a local maternity hospital after normal delivery. First, a 10 ml sample of cord blood was analyzed for communicable diseases, including hepatitis B virus, hepatitis C virus, HIV, cytomegalovirus, and syphilis. The UC was then rinsed twice, and the cord blood was removed during this process. The washed cords were cut into 1 mm^2^ pieces and floated in low-glucose Dulbecco’s modified Eagle’s medium (DMEM) containing 10 % fetal bovine serum (FBS; Stemcell, Vancouver, Canada). The pieces of cord were subsequently incubated at 37 °C in a humidified atmosphere consisting of 5 % CO_2_. Nonadherent cells were removed by washing. The medium was replaced every 3 days after the initial plating. When well-developed colonies of fibroblast-like cells appeared after 10 days, the cultures were harvested with 0.05 % trypsin/ethylenediamine tetraacetic acid (Gibco Life Technologies, Grand Island, NY, USA) and passaged into a new flask for further expansion. UC MSCs used for treatment were subject to passing quality control tests, including immunophenotype identification and analysis of differentiation capacity. Flow cytometric analysis confirmed the cells expressed CD106, CD105, CD90, CD71, CD44, and CD29, but not CD34, CD14, CD3, or CD45 (all of the antibodies and their corresponding isotypes were purchased from BD Biosciences, San Jose, CA, USA). The capacity of MSCs to differentiate along adipogenic and osteogenic lineages was evaluated as described previously [32]. The cells with purity of more than 95 % at passages 3 were used. Before transplantation, the cells were washed and the FBS was removed from the culture medium. For the procedure, a total of 5.2 × 10^7^ cells suspended in 100 ml saline were slowly infused by a heparinized syringe through the cubital vein of the arm over 30 minutes and 1.1 × 10^7^ cells suspended in 10 ml saline were slowly injected intrathecally, after informed consent was obtained from the patient and his family.

## Results

No adverse events associated with transplantation were observed during or immediately after UC-MSCT in the patient.

### Two days post transplantation

Two days after the transplantation, the patient presented less leg numbness and regained muscle strength of the four limbs against gravity. Bladder function improved 2 weeks after the transplantation. The patient was then even able to walk slowly with assistance and he was discharged from hospital in a stable condition with a regimen of prednisone 10 mg/day and the neural nutrition medicine.

### One month post transplantation

At 1-month follow-up after the transplantation, the patient displayed good general condition with leg numbness only. He was able to walk without assistance and his incontinence was replaced by urgency. Control MRI, taken 6 months later after transplantation, showed obvious regression of the previous enhancing lesions. The swelling of the spinal cord was reduced significantly (Fig. 1b).

### Nine months post transplantation

Nine months after first discharge, the patient was readmitted to our center for second progressive leg weakness. He noted rapidly progressive difficulty in rising from low chairs and climbing stairs. Repeated MRI did not reveal a new enhancing lesion involving the spinal cord. He received the same therapy with UC-MSCT, combined with oral prednisone 20 mg/day. The UC was obtained from another healthy mother. Therapy was still well tolerated and his symptoms returned to baseline.

### Eighteen months post transplantation

The patient had been followed up for 18 months since the first transplantation. No severe infections, no local recurrence or distant metastasis of prior tumor, and no second tumor occurred. A follow-up MRI scan did not show new enhanced lesions in the spinal cord (Fig. 1c). In our case, neurological symptoms of the patient developed progressively with middle-dose steroid treatment, and did not improve after 5 days of high-dose steroid administration, so UC-MSCT was then employed. The improvement of the clinical and radiographic results suggested that UC-MSCT might be effective in patients with radiation myelitis. To our knowledge, this is the first report about UC-MSCT in treatment for radiation myelitis.

## Discussion

Theories related to the pathogenesis of radiation-induced CNS lesions fall into two main categories [34, 35]. One category is necrosis due to the death or reproductive sterilization of glial cells and axons, while the other is vascular lesions such as edema, fibrin exudation, and even thrombosis and hemorrhage. Human autopsy studies showed different degrees of nonspecific inflammatory responses, including focal or diffuse infiltration of lymphocytic infiltration, reactive astrocytosis and microgliosis in injured spinal cord. These responses produce and release proinflammatory cytokines such as IL-1 and tumor necrosis factor alpha (TNFα) [36, 37]. In our case, the improvement of neurological signs and symptoms was associated with or resulting from UC-MSCT, since treatment with steroids before the transplantation was ineffective. It is difficult to ascertain the direct role of MSC transplantation in the treatment of radiation myelitis. We hypothesize that the possible mechanistic rationale was mainly based on the abilities of MSCs to promote remyelination, to inhibit toxic inflammation, and to regenerate axons and neurons after they migrate into the lesion site, which requires further clinical and basic investigations for confirmation.

## Conclusion

In summary, this case showed that MSC transplantation might be effective in treatment of radiation myelitis, although the length of follow-up was short. More clinical studies are needed to confirm the efficacy.

## Abbreviations

CNS: Central nervous system
IL: Interleukin
MRI: Magnetic resonance imaging
MSC: Mesenchymal stem cell
NK: Natural killer
TNFα: tumor necrosis factor alpha
UC: Umbilical cord
UC-MSCT: umbilical cord-derived mesenchymal stem cell transplantation
